# The effect of the radio-protective agents ethanol, trimethylglycine, and beer on survival of X-ray-sterilized male *Aedes aegypti*

**DOI:** 10.1186/1756-3305-6-211

**Published:** 2013-07-18

**Authors:** Stacy D Rodriguez, Ramaninder K Brar, Lisa L Drake, Hannah E Drumm, David P Price, John I Hammond, Jacob Urquidi, Immo A Hansen

**Affiliations:** 1 Department of Biology, New Mexico State University, Las Cruces, NM, USA; 2 Department of Physics, New Mexico State University, Las Cruces, NM, USA; 3 Molecular Biology Program, New Mexico State University, Las Cruces, NM, USA; 4 Institute of Applied Biosciences, New Mexico State University, Las Cruces, NM, USA; 5 Department of Biology, University of New Mexico, Albuquerque, NM, USA

**Keywords:** Sterile insect technique, Mosquitoes, *Aedes aegypti*, X-rays, Radioprotectors, Trimethylglycine, Ethanol, Beer, Survival, Longevity, Sterility, Fecundity

## Abstract

**Background:**

Sterile Insect Technique (SIT) has been successfully implemented to control, and in some cases, eradicate, *dipteran* insect populations. SIT has great potential as a mosquito control method. Different sterilization methods have been used on mosquitoes ranging from chemosterilization to genetically modified sterile male mosquito strains; however, sterilization with ionizing radiation is the method of choice for effective sterilization of male insects for most species. The lack of gentle radiation methods has resulted in significant complications when SIT has been applied to mosquitoes. Several studies report that irradiating mosquitoes resulted in a decrease in longevity and mating success compared to unirradiated males.

The present study explored new protocols for mosquito sterilization with ionizing radiation that minimized detrimental effects on the longevity of irradiated males.

**Methods:**

We tested three compounds that have been shown to act as radioprotectors in the mouse model system - ethanol, trimethylglycine, and beer. Male *Aedes aegypti* were treated with one of three chosen potential radioprotectors and were subsequently irradiated with identical doses of long-wavelength X-rays. We evaluated the effect of these radioprotectors on the longevity of male mosquito after irradiation.

**Results:**

We found that X-ray irradiation with an absorbed dose of 1.17 gy confers complete sterility. Irradiation with this dose significantly shortened the lifespan of male mosquitoes and all three radioprotectors tested significantly enhanced the lifespan of irradiated mosquito males.

**Conclusion:**

Our results suggest that treatment with ethanol, beer, or trimethylglycine before irradiation can be used to enhance longevity in mosquitoes.

## Background

Sterile Insect Technique (SIT), an environmentally friendly method of insect extermination without the use of pesticides, has been shown to be a very effective method to control insect species, such as the new world screwworm, *Cochliomyia hominivorax,* the Tsetse fly *Glossina morsitans,* and several species of fruit flies [1-4].

Conceptually established in the 1950′s by Edward Knipling [5-7], SIT has been applied only sporadically and with varying success in experimental mosquito control programs (reviewed in [8]). The development of genetically modified mosquitoes and subsequent genetic control methods such as RIDL (Release of Insects carrying a Dominant Lethal gene), have reawakened the interest in mosquito SIT in the last decade [9-12]. However, sociological and environmental problems with the release of genetically modified organisms (GMOs) make this approach problematic and justify further development of alternative mosquito sterilization methods. For example, a public outcry ensued when the Florida Mosquito Control District announced in 2012 that they were planning to use RIDL mosquitoes to control *Aedes aegypti* populations in Key West. The RIDL project is currently still waiting for approval by the United States Food and Drug Administration (Michael S. Doyle, Executive Director, Florida Keys Mosquito Control District, personal communication, 06/2013). On the other hand, the availability of commercial X-ray sterilization machines [13] makes sterilization using ionizing radiation much more affordable and independent of radioisotope based radiation sources that require extensive safety and disposal measures [14].

Treatment with ionizing radiation damages reproductive cells as well as somatic cells. The main part of spermatogenesis in *Aedes aegypti* takes place during the larval and pupal stages of development [15]. Therefore, to effectively sterilize adult mosquito males, the radiation dose has to be high enough to produce dominant lethal mutations in essentially all spermatozoa. As a consequence, effective doses are generally so high that they reduce the competitiveness of irradiated mosquito males by damaging somatic cells [14].

Several strategies to enhance competitiveness of sterilized males of a variety of different insect species have been developed and many are based on an enhancement of oxidative stress resistance. Oxidative stress resistance can be heightened by an exposure of males to oxidative stress itself prior to irradiation or by administration of antioxidants. For example, hypoxia, induced by oxygen depletion has been successfully used to enhance male sexual performance in Mediterranean fruit flies, *Ceratitis capitata*[16], the Caribbean fruit fly, *Anastrepha suspensa*[17] as well as in *Aedes aegypti*[18]. A handful of unrelated chemicals, DMSO, cysteine, cysteamine, EDTA, and AET have been suggested as potential radioprotectors in mosquitoes (reviewed in [14]).

We chose three potential radioprotectors for this study: lager beer, the beer component N,N,N-trimethylglycine (TMG), and a 5% ethanol solution. These compounds have been shown to significantly reduce radiation injury in mice through unknown mechanisms [19,20]. We evaluated the effect of these three potential radioprotectors on the survival of male *Aedes aegypti*, Yellow Fever mosquitoes, after X-ray irradiation.

## Methods

### Ethics statement

The research plan used for this work involving animals was specifically approved by the Institutional Animal Care and Use Committee (IACUC) at New Mexico State University under approval ID #2011-041. All procedures and care are described in the New Mexico State University Animal Care Facility Standard Operating Procedure and on file in the IACUC office there. All persons involved in animal work successfully completed Animal Welfare Training at New Mexico State University and were specifically trained in protocols used in the research plan. All New Mexico State University IACUC care and protocols follow the NIH guidelines described in Guide for the Care and Use of Laboratory Animals: Eighth Edition, ISBN-10: 0-309-15400-6.

### Mosquito culture

The *Aedes aegypti* Rockefeller strain was obtained from MR4 [21]. Mosquito care and maintenance was performed as described in [22], with chickens (*Gallus gallus*) as blood source. Larvae were reared in pans containing 300 mosquitoes per 2 L of water at 27°C. Larvae were fed ad libitum with commercially available dry cat food pellets (Special Kitty^®^). Five pellets were added per pan and water was changed once after three days. Within 24 hours post-eclosion mosquitoes were separated by gender and placed in a 30 × 30 × 30 BugDorm-1 Insect Rearing Cage (Bugdorm Store, MegaView Science, Taiwan) and placed in an incubation room at 80% humidity and 27°C with a photoperiod of 14 h light and 10 hours darkness.

### Radioprotector treatments

To expose male mosquitoes to the potential radioprotectors, solutions containing these substances were offered as the sole source of hydration 48 hours prior to irradiation in 50 ml flasks with a cotton wick. The following agents were investigated in this study: N,N,N-trimethylglycine (0.08 g/ml, Sigma), ethanol (5% v/v, Sigma), and British organic lager beer (5% APV, Samuel Smith Old Brewery, U.K). 20% w/v sucrose solution (Sigma) was used as a control treatment. Uptake of the various solutions was monitored by visual inspection. We observed male mosquitoes readily taking up all of the liquids offered.

### Mosquito irradiation

X-ray irradiation was performed in a custom built X-ray machine employing a sealed tube. The source was a Jarrel Ash powder diffractometer (Jarre-Ash, MA, USA) running standard Norelco X-ray tubes. The high voltage side was maintained at 40 kV with a current of 10 mA. For each treatment, three groups of 25 mosquitoes were transferred into a 20 ml plastic aspirator collection vial that was inserted into a brass tube for exposure. Mosquitoes were irradiated with an uncollimated unfiltered beam emanating from a Molybdenum target. External shielding was provided by a lead sleeve that covered the assembly. The mosquito housing was water cooled and regulated to a range of 21 to 24°C. The radiation spectrum emanating from the tube was a combination of the Bremsstrahlung background resulting from the 40 kV accelerating potential and the characteristic lines associated with molybdenum. Unirradiated control mosquitoes were placed in the irradiation chamber for the same amount of time and kept under identical conditions with the exception of an applied radiation field. The delivered beam was measured using a Cadmium-Zinc-Telluride Co-Planar detector which operates at room temperature, possess a high detector volume, and has a high resolution. These detectors are typically employed in Nuclear Spectroscopic studies for isotope identification. They have been successfully employed by JU for the characterization of the X-ray energy spectrum prior to performing anomalous scattering. The dose delivered by the beam was determined by measuring the counts delivered in front of absorbing foils. The foils were removed one at a time to avoid saturation of the detector. Saturation occurred in the linear region and we extrapolated the last few foils to zero foil intensity. The results were normalized to the solid angle irradiated during the experiments. This is a standard method for determining the dose delivered by any given intense source.

### Survival analysis

Following radiation treatment, mosquitoes were placed in separate 17.5 × 17.5 × 17.5 cm BugDorm-41515 Insect Rearing Cages and given a 20% sucrose solution. Fatalities were determined every 24 hours until the last mosquito died.

### Evaluating the relationship between irradiation dosage and male sterility

There were three groups of males that were irradiated with different doses of ionizing radiation and one control group. The irradiated groups were given the following absorbed doses: .39 gy, 1.17 gy, or 2.34 gy. The control was placed in the X-ray machine, but received no ionizing radiation. Post radiation treatment mosquito males were placed in a BugDorm-4 (24.5 × 24.5 × 24.5 cm) cage and allowed to recover for 24 hours with access to 20% sucrose solution. After this recovery period, females were added to the cages to achieve a 1:1 sex ratio and kept in the incubation room for 72 hours to allow copulation. The females were then given a blood meal by putting a restrained chicken on top of the cage for 30 minutes. Thirty six fully engorged female mosquitoes were taken from each group and placed into individual 50 ml falcon tubes containing a wet cotton ball at the base of the tube, topped by filter paper for egg deposition. A total of 144 females were used for this experiment, 12 groups with 12 blood fed mosquitoes per group. Females were removed after six days. The eggs were collected and counted for each individual female. Next, the eggs were air dried and stored for six days in the insect chamber. Then, the eggs were counted and mixed en mass for each group. Three groups of one hundred eggs were randomly selected for hatching. Egg viability was determined three days after hatching by counting larvae. Three biological replicates were analyzed.

### Sterility assessment for radioprotector experiment

Mosquitoes were separated by sex post eclosion, twice daily to ensure virginity of females. The mosquitoes were separated into 5 groups of 50 male mosquitoes. Males were given sugar for 24 hours post eclosion and then administered one of the following treatments for 48 hours prior to irradiation: .08 g/ml TMG, 5% ethanol, lager, or 20% sucrose. The males were given an absorbed dose of 1.17 gy of ionizing radiation and given 24 hours to recover on sugar before being placed in a mating cage with females at a 1:1 ratio. They were allowed to mate for 72 hours before the females were given a blood meal. Twenty fully engorged females from each cage were placed in separate egg laying chambers. The females were given 6 days to lay eggs. After six days, the eggs were then mixed together (4–5 females eggs were mixed as a group) and 100 eggs from each group were hatched. Each treatment had 3 groups, except for the 20% sucrose irradiated group, due to the limited number of females that laid eggs; instead the 20% sucrose treatment contained two groups. Each group of 100 eggs was placed in 100 ml to hatch using a vacuum then transferred to a pan with 500 ml of water to hatch for 3 days. The larvae from each batch were then counted. Three biological replicates were analyzed.

### Statistical analysis

Survival differences between treatments for the ethanol and the radioprotector experiments were assayed by a log-rank test performed using the ‘survdiff’ function in the ‘survival’ library for R [23]. P < 0.05, was used for significance testing. Pair-wise treatment comparisons were made between all treatments. The Holm-Bonferroni method was used to adjust alpha and reduce type I error [24]. For the ethanol survival assay, life time survival was assayed. For the radioprotector experiment, survival was divided into one and two week sections to reflect likely survival in nature. Next, Hedges’ g* was calculated for each treatment in comparison to the unirradiated control and the sucrose only treatment for days one through fourteen to test for effect size [25]. Means were generated through one and two week periods. Lastly, ANOVAs with planned contrasts were performed on rank transformed data to determine the day at which each treatment became significantly different from the unirradiated and sucrose controls.

## Results

### The effect of ethanol on mosquito survival

**Figure 1:**
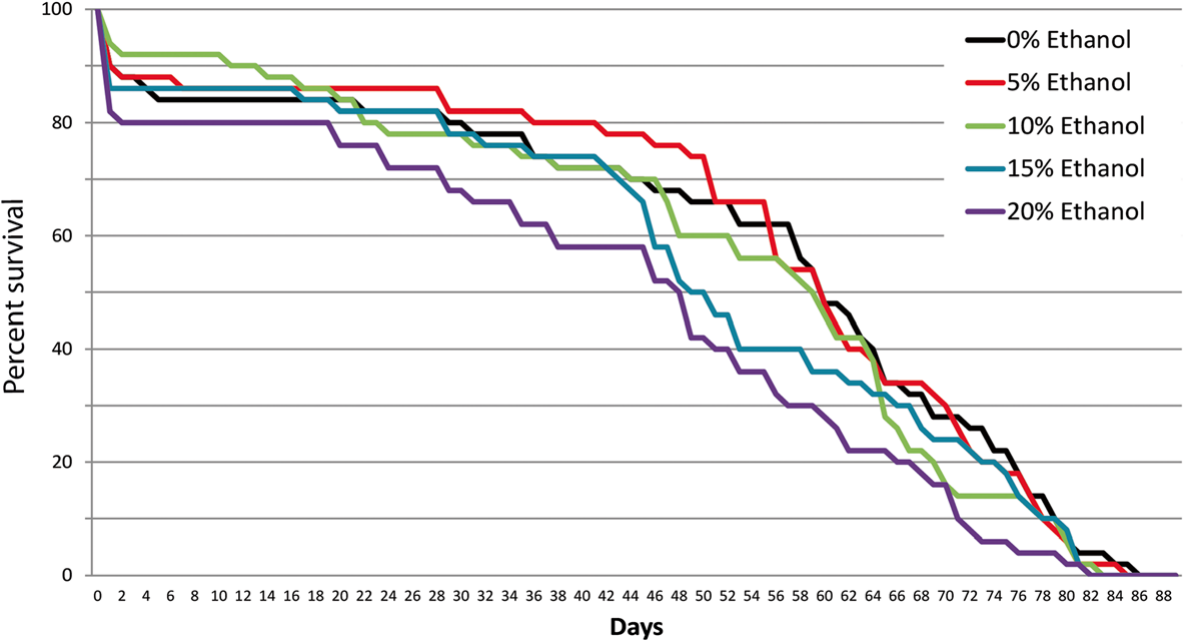
**Survival plots for groups of 50 male *Aedes aegypti *mosquitoes that were continuously treated with different concentrations of ethanol during their adult life and control mosquitoes. **The percent survival is shown each day after hatching. The Log-Rank test showed a significant difference between the 0% and 20% ethanol treatment as well as a difference between the 5% and 20% ethanol treatments.

To measure the effect of long-term exposure to ethanol on the lifespan of male *Aedes aegypti* mosquitoes we evaluated the survival curves of groups of unirradiated mosquito males that were kept with 20% sucrose solution containing varying concentrations of ethanol for their complete adult life. These males were housed without females in BugDorm-4 cages in groups of 50. Survival plots for these groups are shown in Figure 1. We found a significant negative effect on survival in the groups treated with the highest concentration of ethanol (20%) and no significant effect with the lower concentrations. Fifty percent mortality was reached at an age of approximately 60 days in the groups treated with lower concentrations of ethanol. The last male from the control group died at an age of 86 days.

### Effects of male X-ray sterilization on female egg numbers

**Figure 2:**
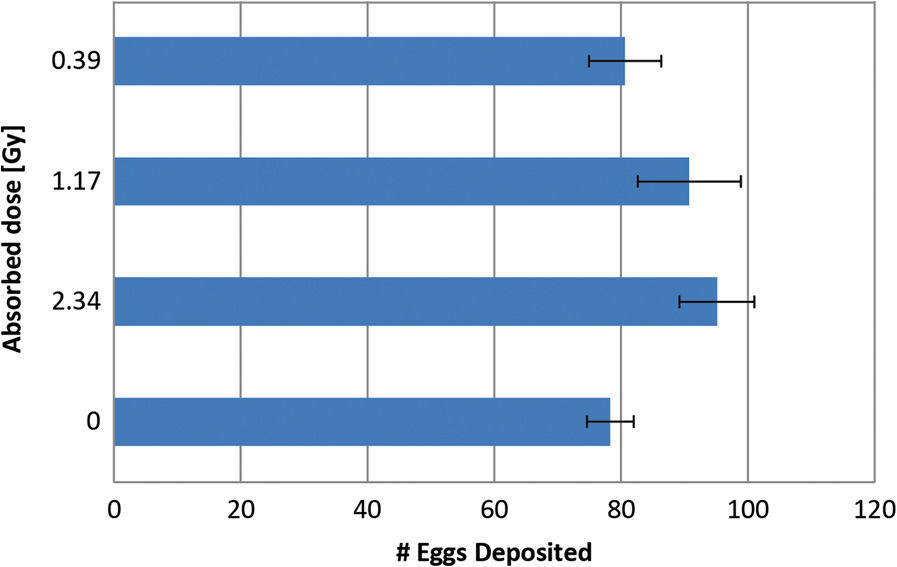
**Effects of male X-ray sterilization on female egg numbers. **Three biological replicates were analyzed for each treatment. The females that mated with irradiated males did not show a reduction in fecundity based on one-paired T-test analysis.

To address concerns about the sperm quality of irradiated mosquito males, we tested if females mated with irradiated males produce equal numbers of eggs compared to females that were mated with unirradiated males (see Figure 2). We did not find significant differences in egg numbers between the experimental and control groups.

### X-ray sterilization effectiveness

**Figure 3:**
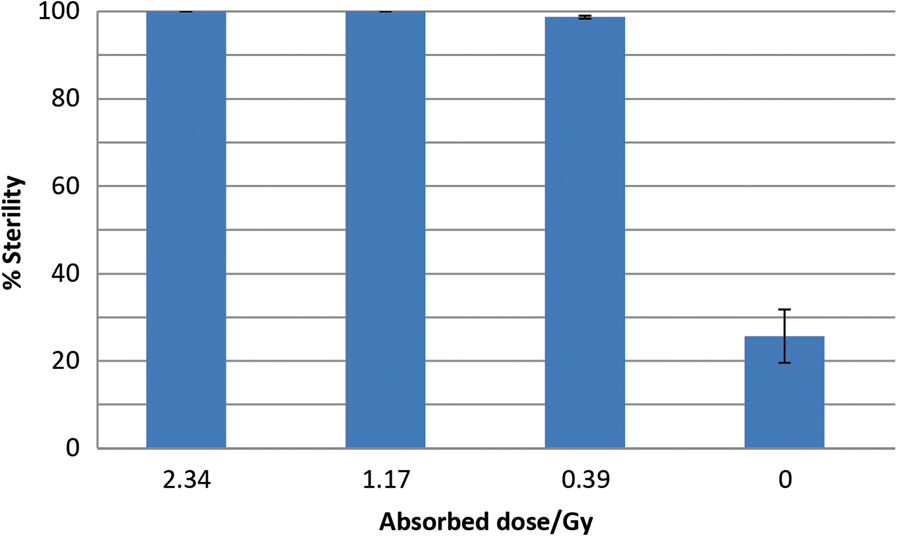
**X-ray sterilization effectiveness. **Effectiveness was demonstrated by egg viability assay. Three biological replicates were analyzed.

The results of our assessment of sterilization effectiveness are shown in Figure 3. Eggs that were laid by females that copulated with males that were irradiated with an absorbed dose of 2.34 gy and absorbed dose of 1.17 gy were 100% +/- 0% unviable. Irradiation with an absorbed dose of .39 gy resulted in less than 1.33% +/- .0033% egg viability. Females mated with unirradiated control mosquitoes had over 74% +/- .0612% egg viability.

### Effect of radioprotector treatment on male survival after X-ray sterilization

**Figure 4:**
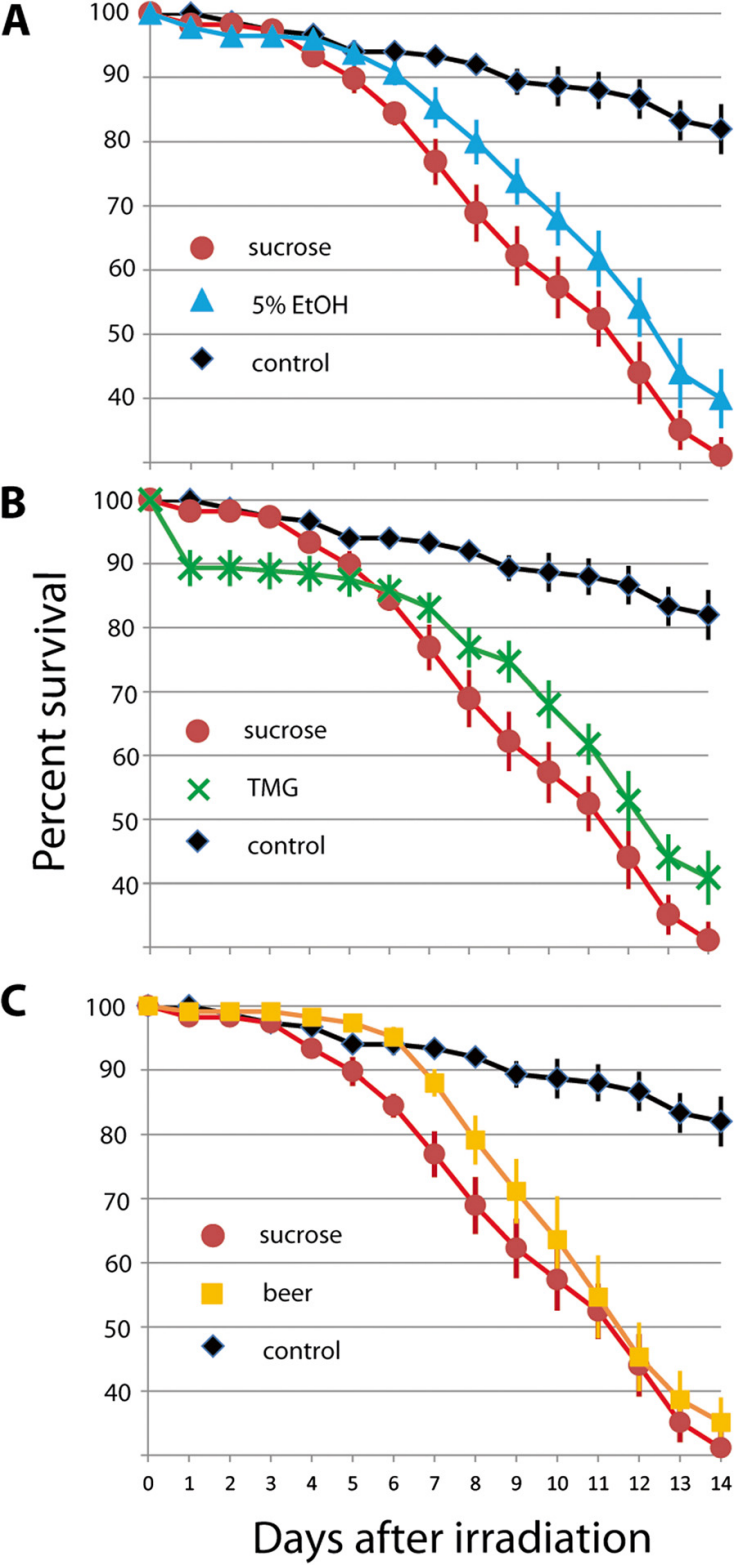
**Effects of radioprotectors on male *Aedes aegypti *longevity after irradiation. **Nine biological replicates were analyzed. Survival plots shown are for **(A) **5% ethanol, **(B) **trimethylglycine (TMG), and **(C) **lager treatments, each compared to the unirradiated and sucrose irradiated control groups. All treatments show significant differences between the unirradiated control and all other treatments through both one and two week time periods (p < 0.05). Ethanol, lager, and TMG treatments all show a significant increase in survival over one or both of the time periods as compared to the sucrose controls. See text for additional results.

The survival curves of irradiated male mosquitoes treated with different potential radioprotectors or sucrose as well as unirradiated males are shown in Figure 4. Through both one and two week periods the unirradiated controls were significantly different from all other treatments (p < 0.05 see Table 1). Mean effect size through one week shows a different pattern with the lager treatment showing little to no difference from the unirradiated control (Hedges’ g* 0.1 with the lager treatment showing higher survival on average). The 5% ethanol group showed a moderate effect (Hedges’ g* 0.5 with the control treatment showing higher survival on average). All other comparisons with the control show large effects of decreased survival with irradiation (Hedges’ g* > 0.9).

**Table 1:** Statistical analysis

**Treatments**	**Through week 1**		**Through week2**		**Average Hedges’s g**	
	**Chi-Square**	**p value**	**Chi-Square**	**p value**	**Days 1-7**	**Days 1-14**
Control vs. Ethanol	8.3	0.004	72.3	<0.001	-0.52	-1.38
Control vs. Lager	4.1	0.042	90.1	<0.001	0.12	-1.16
Control vs. Sucrose	24.8	<0.001	110	<0.001	-0.89	-2.11
Control vs. TMG	12.8	<0.001	70.9	<0.001	-1.35	-1.96
Sucrose vs. Ethanol	5.2	0.023	5.8	0.016	0.32	0.54
Sucrose vs. Lager	10.2	0.001	1.9	0.171	1.02	0.69
Sucrose vs. TMG	2	0.157	5.1	0.023	-0.57	0.11
Ethanol vs. Lager	0.9	0.354	1.3	0.254	-0.60	-0.14
Ethanol vs. TMG	0.6	0.436	0	0.974	0.87	0.45
Lager vs TMG	2.7	0.098	1.3	0.263	1.38	0.54

Pair-wise comparisons over all treatments for the survival analysis through week one and through week two are presented. Average hedges’ g for days 1 through 7 and 1 through 14 of the experiment are also presented with positive values representing increased survival as compared to the unirradiated control or sucrose irradiated treatments. The comparisons for the ethanol, lager, and TMG treatments are presented as being positive if the ethanol treatment group survives better (Ethanol vs. Lager, and Ethanol vs. TMG) or that the lager shows higher survival (Lager vs. TMG).

All three potential radioprotectors tested resulted in a significant improvement in male survival after radiation as compared to the sucrose irradiated control over at least one time period (Table 1). The 5% ethanol treatment group showed a significant increase in survival through both one (p = 0.023) and two week (p = 0.016) time periods. Treatment with TMG showed no difference through week one (p = 0.16), but increased survival through two weeks (p = 0.023). The lager treatment group showed the opposite pattern, with increased survival through week one (p = 0.001), but not through two weeks (p = 0.17). Mean effect size showed a small (Hedges’ g* 0.3) to moderate (Hedges’ g* 0.5) effect from ethanol treatment through time. The TMG treatment showed a moderate effect through week one with decreased survival (Hedges’ g* -0.6) and no effect through two weeks (Hedges’ g*0.1). The lager treatment showed a large increase in survival effect (Hedges’ g*1.0), which decreased to a moderate effect (Hedges’ g* 0.7), through two weeks. In general, the three radioprotectors showed similar levels of protection to each other for the survival analysis through both one and two week time periods (no significant differences). The average effect size measure tends to show that lager does better than the ethanol, which does better than the TMG especially through the first week (Table 1).

### Effect of radioprotector treatment on sterilization effectiveness

Based on egg viability, Lager treated irradiated mosquito males were 99.67% +/- .0033 sterile. Sucrose treated irradiated male mosquitoes were determined to be 100% +/- 0 sterile. The sterility for males treated with TMG was 99.67% +/- .0033. The ethanol treated males remained 100% +/-0 sterile. Eggs from non-irradiated males mated with females had an 81.67% +/- .0611 hatch rate.

## Discussion

The easiest and most effective way to battle mosquito-borne diseases like malaria, Dengue, Yellow Fever, or filariasis is by breaking the pathogen transmission cycles by controlling mosquito populations. Several effective approaches for mosquito population control have been developed, for example, indoor-residual spraying, insecticide-treated bed nets, *Bacillus thuringiensis* toxin-based larvicides, etc. The aim of this study was to explore new ways to improve mosquito SIT, which we think has the potential to become a valuable addition in the repertoire of mosquito control tools available. To this end we tested the effect of three potential radioprotectors on mosquito male longevity.

At the onset of this study, we addressed the concern that some of the radioprotectors we planned to test might be toxic to male mosquitoes and reduce their lifespan – an undesirable effect for SIT. Since two of the potential radioprotectors tested were alcoholic solutions, we started by testing the effect of long-term alcohol exposure on male mosquito lifespan. We found that long-term exposure to 5, 10% and 15% alcohol had no significant effect on mosquito survival (Figure 1), while the highest concentration (20% vol/vol) was only modestly detrimental. A string search on vectorbase.org [26,27], revealed that *Ae. aegypti* has more than a dozen genes encoding potential alcohol dehydrogenase proteins and our experiment suggests that male mosquitoes have the ability to metabolize ethanol quite effectively. We concluded that long-term exposure to ethanol at concentrations of 5% or below is not harmful for male survival in mosquitoes. It would be interesting to test if long-term exposure to even lower concentration of ethanol result in prolonged life span as has been shown in the *C. elegans* model system [28].

Male mosquitoes in abstinence under laboratory conditions with permanent access to carbohydrates showed a surprisingly long lifespan compared to male mosquitoes in standard laboratory mixed sex cultures, where they usually die within the second and the third week post eclosion. The last control mosquito male died 86 days after hatching. This number compares to large female mosquitoes with permanent access to sugar water that have been shown to have a maximum life span of up to 90 days [29]. However, we propose that it is very unlikely that male *Ae. aegypti* reach comparable ages in nature because of natural stressors such as temperature, humidity, predators, and parasites that are absent in the laboratory environment. In fact a study in the Tucson area showed that only 9% of all *Aedes aegypti* females trapped were 15 days or older [30]. Female mosquitoes do not lay eggs when not inseminated (27). Male seminal fluid proteins cause behavioral and physiological changes in the female and thereby regulate reproduction (reviewed in [31]). Therefore, we tested the effect of mating females with irradiated males vs. unirradiated males on egg number as an indirect measure of seminal fluid quality. We found that neither egg numbers (Figure 2) nor the total percentage of females that laid eggs after a blood meal (data not shown) differed between the two groups. We conclude from this experiment that there is no relevant difference between the seminal fluid of irradiated males compared to unirradiated ones when it comes to inducing vitellogenesis and egg development in females.

The radiation dosage is important for SIT implementation in the field. A balanced dosage should be used that provides a high level of sterility and at the same time does minimal somatic damage to the male. We found that treatment with 1.17 gy from an unfiltered, uncollimated molybdenum x-ray source resulted in complete sterility in males (Figure 3). We therefore chose this dose for all subsequent experiments. The dosage necessary to achieve complete sterility in our study is much lower than dosages reported in other studies [12]. Radiation doses and absorbed doses are related to one another through a quality factor that appropriately scales the radiation being employed to account for the effectiveness of energy transfer in biological systems. For X-rays and gamma rays the quality factor is 1.0 and so the radiation dose is the same as the absorbed dose. The units for absorbed dose presented in the manuscript were in Grays as is standard in the literature. The reason for the lower dose is the use of longer wavelength radiation than is typical in the literature. This results in a more effective transfer of energy to the organism. The details of this are the focus of an upcoming manuscript where we report the relationship between X-ray wavelength and sterility and survival.

Identification of radioprotective substances and their application has been a focus of interest since the start of the nuclear area. Radioprotectors are of interest in the context of accidental exposure to ionizing radiation but also in the field of medical radiation biology and space exploration [32]. A plethora of agents have been identified and characterized (reviewed in [33]) and a handful of different agents have been tested in relation to SIT. We chose the three radioprotectors tested in this study because they have been shown to effectively protect mice and human lymphocytes from radiation damage [20,34], and to our knowledge have never been tested in mosquitoes. Ethanol and lager beer are easily available in most countries around the world and TMG is sold as a food supplement for relatively low costs. It must be noted, that in contrast to the relatively “simple” ethanol and TMG-solutions we used to treat our male mosquitoes, beer is a highly complex solution which contains a large number of different compounds [35]. Interestingly, treatment with all three agents resulted in improved survival in mosquito males after radiation exposure (see Figure 4). All three agents can therefore be considered as radioprotectors. Notably and in contrast to the other treatments, TMG-treatment resulted in relatively high 1-day mortality of 10%. TMG might have a higher acute toxicity to mosquitoes than ethanol or beer and more experiments are necessary to determine the dose response curve for this chemical. The mode of action of all three radioprotectors in the mosquito system is unknown, however, ethanol and alcoholic beverages have been shown to increase oxidative stress in eukaryotic cells [36-38]. TMG is a methyl donor in methylation reactions and might have antioxidant properties.

## Conclusion

In conclusion, we propose that treatment with one of the three radioprotectors can be easily integrated in most SIT protocols at low additional costs and with a significant improvement of male survival in the critical time period after release. This can lead to reduced SIT costs because less sterile males have to be produced to achieve the same impact.

## Abbreviations

SIT: Sterile insect technique; TMG: Trimethylglycine.

## Competing interests

The authors declare that they have no competing interests.

## Authors’ contributions

SR, IH, and JU designed all the experiments including: the sterilization and fecundity experiment, the radioprotector evaluation experiment, and the ethanol longevity experiment. SR and LD carried out the mosquito culture. LD and SR prepped radioprotector solutions for treatments. SR and LD separated males and females ensuring virginity that was required for the sterility experiment. SR and LD separated males from females to irradiate. SR, LD, HD helped record longevity and maintain mosquitoes post irradiation. JU and RB irradiated mosquitoes, performed the dosimetry and maintained the X-ray machine. DP and JH performed statistical analysis. All authors read and approved the final version of the manuscript.
